# Characterization of chloroplast genome of *Eleusine coracana*, a highly adaptable cereal crop with high nutritional reputation

**DOI:** 10.1080/23802359.2021.1972863

**Published:** 2021-08-31

**Authors:** Li-Ying Feng, Li-Zhi Gao

**Affiliations:** Institution of Genomics and Bioinformatics, South China Agricultural University, Guangzhou, China

**Keywords:** *Eleusine coracana*, chloroplast genome, Poaceae, phylogeny

## Abstract

*Eleusine coracana* (L.) Gaertn. is a kind of highly adaptable cereal crop with a high nutritional value with the reputation of ‘black pearl’. In this study, we sequenced, assembled and characterized the complete chloroplast genome of the grass species. The circular genome of *E. coracana* was 135,137 bp in length, which comprised two inverted repeat (IRa and IRb) regions of 20,919 bp in length separated by a large single copy (LSC) region of 80,663 bp and a small single copy (SSC) region of 12,636 bp. The total GC content of the *E. coracana* chloroplast genome was ∼38.13%. A total of 108 functional genes were predicted, including 76 protein-coding genes, 28 tRNA genes, and four rRNA genes. Our phylogenomic analysis of all protein-coding genes further revealed that *E. coracana* is closely related to *Bouteloua curtipendula* and *B. gracilis*, and they are together positioned in the subfamily Chloridoideae clade of the grass family.

*Eleusine coracana* (L.) Gaertn., an annual herbaceous grass, is a staple food crop in the semi-arid tropics. Its grains can be utilized to brew beer, and its straw can be used for the preparation and papermaking raw materials (Hanna 1995). In China, *E. coracana* has long served as a traditional Chinese medicine, such as the treatment of stomach disease. It is a good medicinal plant with high nutritional and antioxidant capacities (Kumar et al. 2016; Sharma et al. 2017). *E. coracana* is rich in calcium, fiber, and iron, with excellent malt quality but low glycemic index (GI), and thus, it is the preferred food for diabetic patients (Pradhan et al. 2010).

In this study, *E. coracana* plants were collected in the suburbs of Guangzhou City (22° 48′ 13″ N, 113° 33′ 25″ E), Guangdong Province, China. A specimen was deposited at SCAU (the herbarium of the College of Agriculture, South China Agricultural University https://nxy.scau.edu.cn, Li-Zhi Gao, SCAUgenomics@163.com), China, under the voucher number SCAU 2020180. The fresh and healthy leaves were sampled and then dried with a silica gel drying method. The chloroplast genomic DNA was extracted using the improved method (CTAB) (Shi et al. 2012). For the genome sequencing, ∼3.4 Gb of Illumina sequencing data with 250 bp insert size were generated on the HiSeq X Ten platform. Using the closely related species, *E. coracana* (GenBank accession number: NC_030486) as a reference, the obtained clean reads were then assembled using GetOrganelle v1.5 (Jin et al. 2020). Subsequently, gene prediction and annotation were performed by CPGAVAS2 (Shi et al. 2019).

The complete chloroplast genome of *E. coracana* was 135,137 bp in size, comprising two inverted repeat regions (IRs) with a total of 41,838 bp in size, which are split by a large single copy (LSC) with 80,663 bp and small single copy (SSC) with 12,636 bp in length. The chloroplast genome contained 108 functional genes, including 76 protein-coding genes, 28 tRNAs, and four rRNAs, among which nine genes had introns. Most of the genes are single-copy genes. However, *ycf3* contained two introns in the *E. coracana* chloroplast genome, and an intron was observed in the nine protein-coding genes (*rpl2*, *rpl16*, *rps12*, *rps16*, *ndhA*, *ndhB*, *petB*, *petD*, *atpF*) and three tRNA genes. A total of 18 genes were repeated in the IR regions, including four rRNA genes, six protein-coding genes, and eight tRNA genes. The overall GC content of the *E. coracana* chloroplast genome was ∼38.13% with the corresponding values of 36.09%, 32.34%, and 44.00% in the LSC, SSC, and IR regions, respectively.

To determine the phylogenetic position of *E. coracana* in the grass family, 34 grass chloroplast genomes together with *Cyperus rotundus* from Cyperaceae were separately downloaded from GenBank. Phylogenomic analysis was performed by incorporating the *E. coracana* chloroplast genome obtained in this study. All protein-coding gene sequences were aligned with MAFFT 7.409 (Katoh et al. 2002). Using *C. rotundus* as outgroup phylogenetic tree was reconstructed using the maximum-likelihood method using RAxML (Stamatakis 2014) based on 1000 bootstrap replicates. Our results indicated that the 35 examined grass species were evidently clustered into the 12 subfamilies of Poaceae with strong bootstrap supports (Figure 1). *E. coracana* is closely related to *Bouteloua curtipendula* and *B. gracilis* and is further grouped with the one other grass species from Chloridoideae with strong bootstrap supports.

**Figure 1.**
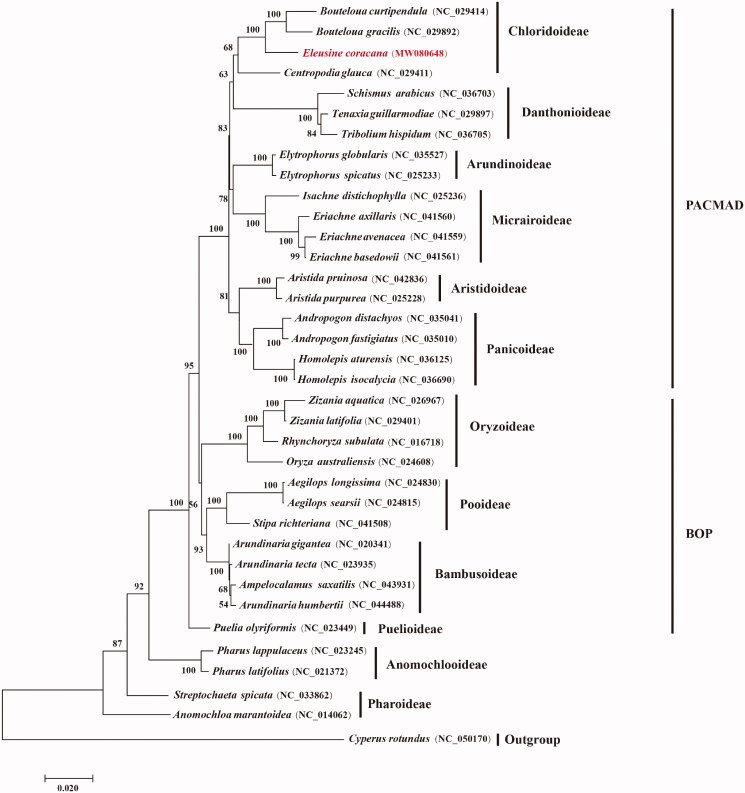
Maximum-likelihood phylogenetic tree based on all protein-coding genes of the 35 grass complete chloroplast genomes using *Cyperus rotundus* as outgroup. Bootstraps values (1000 replicates) are shown at the nodes.

## Data Availability

The genome sequence data that support the findings of this study are openly available in GenBank of NCBI at https://www.ncbi.nlm.nih.gov under the accession number MW080648. The associated BioProject, SRA, and Bio-Sample numbers are PRJNA667839, SRR15049124, and SAMN16386298, respectively.
